# Report

**Published:** 1885-09

**Authors:** 


### PROCEEDINGS.

### Report.

The Minnesota Dental Society held the second annual meeting in Curtiss Hall, Minneapolis, July 31st, August 1st and 3rd.

First day, routine business, afternoon session the address of President L. W. Lyon, St. Paul, was delivered.

Dr. Knight, of Minneapolis then read his essay on Pulpless Teeth.

In regard to filling roots while they were bleeding at the apex, Dr. Clements says he favored doing it immediately after removal of the nerve and as soon as the blood stopped flowing. For this he used oxy-chloride of zinc as it formed a cicatrix which prevented further hemorrhage, thus not leaving time for any discharge to form.

Dr. Avery said he always preferred to wait from two to seven days after the removal of the nerve before he filled the canal.

Dr. Knight related one case where thinking the hemorrhage had ceased, he filled the root, but inflammation ensued, and on removing the filling there was a free flow of blood, after further treatment the case was made comfortable and satisfactory.

Dr. A. T. Smith said if the root was filled before the flow of blood had entirely ceased that it would continue for a time to flow and by its presence or pressure cause inflammation of the membrane, abscess, etc. He always waited several days after the removal of a pulp before filling a root, had received much help on the subject from Dr. Farrar, he favored as free opening as possible in root canal, but had never had good success with the Talbot Reamers, this he ascribed later, to a lack of knowledge in regard to the proper method of using them.

Dr. Bailey had had poor success in drilling roots and was bothered most by anterior roots of lower molars, and in many cases his results had not been satisfactory—and to avoid passing the apex he gauges his drill by a small piece of rubber dam on the shank. For filling roots he generally used gutta-percha cylinders, having first wet them with chloroform.

Dr. Talbot of Chicago being present, was called on and explained his set of reamers, showing their uses, and how they were often abused through ignorance, said he was glad to have heard the adverse as well as the favorable criticisms. Thinks oxychloride of zinc the only proper root filling, owing to its caustic and preservative effect on animal matter sealing a root tight, and more fully preventing formation of gases; the only objection to it being, the trouble it causes when forced through the apex.

Dr. Williamson often uses for filling nerve canals a paste of oil of cloves, oxide of zinc and carbolic acid, this being hard enough to produce for him satisfactory results. The meeting then adjourned.

SECOND DAY—MORNING.

The first on the programme was Dr. Hale's paper on the Minute Anatomy of the Teeth. As to why creosote was used. Dr. Marshall said it was a germicide and escharotic, and although other remedies possess these properties, it seems to be better combined for dental uses.

Dr. J. H. Spaulding referred to Dr. Miller's zone of softened dentine around the decay of a cavity caused by micro-organisms.

Dr. J. R. Patrick, of Bellevue, Ill., said he thought micro-organisms were *not* the cause of decay, as experimentors have never shown whether it is decay or organisms that come first—if a little decay is left in a tooth a filling with perfect margins put over it stops the decay, which it should not do were it full of micro-organisms.

Dr. Marshall said he had seen cases where decay did progress under a filling, enough to expose a pulp, why, he could not say, only it was there, whether decay or only absorption.

Dr. Patrick thinks in a few years many writers will change their views on the subject of micro-organisms.

Very recently a thorough investigation by three experimentors with the microscope proved that instead of being three kinds of bacteria it was only the three forms of *one* in its development.

If this is the result of the past two weeks what results will future investigations show?

Dr. Marshall's paper was then read. (See page 453.)

Considerable discussion was had as to the full action of per oxide of hydrogen on abscesses, etc.

Dr. Marshall thought a union took place between some of the elements, producing the gas sufficient to cleanse the cavity of the abscess, said Dr. Harlan's theory of medical effect was that the extra atom of oxygen unites with the sulphur and forms sulphuric acid, which acted as a slight caustic on the pyogenic membrane, thus cleansing, and tending to prevent the formation of pus.

He himself claimed chief its value as a germicide and cleansing agent.

AFTERNOON SESSION.

Dr. Biglow's paper. (See page 453.)

Dr. Weeks said he thought parents should be taught not to picture the dentist as such a terrible object to the children. Though such remarks are often made thoughtlessly, they leave an impression on the child's mind that it often requires years to eradicate, if it is ever done.

Dr. Cruttenden thought deaf children had less dread of dental operations because they did not hear these thoughtless remarks of "would be martyrs."

Dr. Jenison said he thought there were many dentists who needed educating in regard to treatment of children. We should be kind, firm and honest with our little patients and *never* under *any* circumstances deceive them, when they will often prove our best patients and strongest friends. To regain the confidence of a child who has been decieved by a dentist is no easy task.

Dr. Bailey mentioned several cases in practice, where by perseverance and kindness he had marked success with very nervous and frightened children, and although force *may* sometimes be necessary, it is not often.

Dr. Marshall.—A dentist who will deceive a child is a good example of "the wickedest alive," Thinks children if properly treated often endure more than adults; said in most cases his success with children was better if the parents were in the reception room instead of by the chair, a dentist's sympathy and judgment being of greater value.

Dr. Patrick thought that a dentist should love children to succeed well with them, for the little ones know more that they get by intuition than any one gives them credit for. Under no circumstances let coaxing or pleading of parents or patient lend us to do what we feel to be wrong, for we are seldom excused from bad results.

Dr. Spaulding, of St. Louis, said, dentists as a class dread dental operations as much as, or more than others. He had submitted to surgical operations that he dreaded much less than having some teeth treated and filled, so what are we to expect of our patients, and to deceive a child is a blunder and a crime.

It should be the study of every dentist to be as painless as possible. In some cases it is better not to be as thorough with young children's teeth as we might desire, than it would be to make them submit to more severe operation. In twenty years he has not extracted a tooth for a child, except to allow room for regulation, and thinks it unnecessary and cruel in simple cases of toothache. Eliminate extracting and you remove much dread from a child's mind, and gain a strong point.

Dr. Jenison's paper. (See page 458.)

Dr. Renolds (Wis.) spoke of his method of giving anæsthetics on a tilting tube prepared for the purpose, and said he had very good satisfaction in using ether and gas in combination. Always gave a stimulant previous to administering any anæsthetic. After some discussion by others the meeting adjourned for a recess.

Dr. Martindale's paper:

Dr. Jenison said that, like others, his experience with Cocaine had been quite variable, but had derived the most satisfactory results. Apply ligatures where cavities extended high on the tooth, where gums were very tender, and in removing tartar that was under the gums.

Dr. Marshall related his experience with the drug from its introduction, and though he did not claim that it was infallible, he had derived much satisfaction from it.

Dr. Frank Allport was called on and said Cocaine is one of those remedies that are constantly appearing in medicine and dentistry, that flash across the sky like a meteor, and at last drop into their proper niche—so would it be with Cocaine. It has its place and is a most valuable agent, but is not a "cure all." Thinks the best results are obtained on mucous and submucous, and raw surfaces.

MONDAY.

Clinic:

Dr. Martindale,—Cocaine.
Dr. Markline,—Electric Mallet.
Dr. Murray,—Contour Filling.
Dr. George Brown,—Bridge Work.
Dr. Patrick,—His Crown Machine.

AFTERNOON.

Election of officers:

*President*, Dr. F. A. Williamson, Red Wing.
*Vice-President*, Dr. C. M. Bailey, Minneapolis.
*Recording Secretary*, M. G. Jenison, Minneapolis.
*Corresponding Secretary*, C. H. Goodrich, St. Paul.
*Treasurer*, Dr. H. M. Reed, Minneapolis.

Adjourned. C. M. BAILEY, *Sec.*

